# Progesterone suppresses the invasion and migration of breast cancer cells irrespective of their progesterone receptor status - a short report

**DOI:** 10.1007/s13402-017-0330-z

**Published:** 2017-06-26

**Authors:** Mukul Godbole, Kanishka Tiwary, Rajendra Badwe, Sudeep Gupta, Amit Dutt

**Affiliations:** 1 Integrated Genomics Laboratory, Advanced Centre for Treatment, Research and Education In Cancer, Tata Memorial Centre, Navi Mumbai, 410210 India; 2 Training School Complex, Homi Bhabha National Institute, Anushakti Nagar, Mumbai, India; 3 Department of Surgical Oncology, Tata Memorial Centre, Tata Memorial Hospital, Mumbai, 400012 India; 4 Department of Medical Oncology, Tata Memorial Centre, Mumbai, 400012 India

**Keywords:** Breast cancer, Progesterone, Cell invasion, Cell migration, Phosphoproteomics, Metastasis

## Abstract

**Purpose:**

Pre-operative progesterone treatment of breast cancer has been shown to confer survival benefits to patients independent of their progesterone receptor (PR) status. The underlying mechanism and the question whether such an effect can also be observed in PR negative breast cancer cells remain to be resolved.

**Methods:**

We performed proteome profiling of PR-positive and PR-negative breast cancer cells in response to progesterone using a phospho-kinase array platform. Western blotting was used to validate the results. Cell-based phenotypic assays were conducted using PR-positive and PR-negative breast cancer cells to assess the effect of progesterone.

**Results:**

We found that progesterone induces de-phosphorylation of 12 out of 43 kinases tested, which are mostly involved in cellular invasion and migration regulation. Consistent with this observation, we found through cell-based phenotypic assays that progesterone inhibits the invasion and migration of breast cancer cells independent of their PR status.

**Conclusion:**

Our results indicate that progesterone can inhibit breast cancer cell invasion and migration mediated by the de-phosphorylation of kinases. This inhibition appears to be independent of the PR status of the breast cancer cells. In a broader context, our study may provide a basis for an association between progesterone treatment and recurrence reduction in breast cancer patients, thereby providing a lead for modelling a randomized in vitro study.

**Electronic supplementary material:**

The online version of this article (doi:10.1007/s13402-017-0330-z) contains supplementary material, which is available to authorized users.

## Introduction

Metastasis is a major cause of recurrence in breast cancer patients [1]. As a standard mode of treatment, patients with metastatic breast cancer are subjected to adjuvant hormonal therapy [2]. Also, pre-operative progesterone treatment has been shown to reduce the recurrence rate among node-positive patients, independent of their progesterone receptor (PR) status [3]. While the invasion and migration of breast cancer cells play major roles in establishing metastases [4, 5], in vitro cell-based observations on the effect of progesterone, restricted to PR-positive cells, have corroborated the clinical observations [6–8]. As yet, however, the effect of progesterone on the invasion and migration of PR-negative breast cancer cells remains to be systematically explored [9].

The metastatic nature of breast cancer cells is known to be affected by multiple molecular factors, including the activation of protein kinases [10]. The protein kinases EGFR, AKT or FAK have, for instance, been found to activate the processes of migration and invasion of breast cancer cells [11, 12]. In addition, it has been found that these kinases may act synergistically and that abrogating their activation may decrease the invasive capacity of breast cancer cells [13]. Also, pathways downstream of these kinases may serve to restrain cell invasion and migration [10]. Although these kinases have been found to be affected by steroid hormone receptors [14], it remains to be explored whether they mediate the responses to progesterone in breast cancer cells.

To address the question whether progesterone can regulate cellular migration and invasion of breast cancer cells independent of their PR status, we selected a panel of breast cancer-derived cell lines with different PR statuses. Next, we performed a phospho-proteomic screening of kinases associated with migration and invasion using a human proteome phospho-kinase array platform, and studied their phosphorylation status after treating the respective cells with progesterone. Our cell-based phenotypic and biochemical analysis results suggest that progesterone may mitigate the invasion and migration of breast cancer cells, irrespective of their PR status.

## Materials and methods

### Breast cancer-derived cells

The BT474, T47D, MCF7, ZR-75-1, MDA-MB-231 and BT-549 breast cancer-derived cell lines were obtained as a gift from Dr. Slamon’s Laboratory (Department of Medicine, UCLA, USA). The cell lines were authenticated by DNA short tandem repeat (STR) profiling using the Promega GenePrint 10 system in conjunction with the GeneMarker HID software tool and the ATCC database. The cells were tested for mycoplasma and, if necessary, made mycoplasma-free using an EZKill Mycoplasma Removal reagent (HiMedia). BT474, T47D, MCF7 and MDA-MB-231 cells were cultured in DMEM medium (Gibco), whereas ZR-75-1 and BT-549 were cultured in RPMI-1640 medium (Gibco). BT-549 cells were supplemented with 0.023 IU/ml insulin. All culture media were supplemented with 10% (*v*/v) FBS (Gibco), 2.5 mg/ml Amphotericin-B (Abbott) and 1.25 μl/ml Gentamycin (Abbott). The cells were cultured at 37 °C in a 5% CO_2_ incubator. The PR/ER/Her2 receptor statuses of all the cells as reported in [15] were validated by reverse transcriptase-PCR.

### Progesterone treatment

Cells were grown to 70–80% confluence and then serum starved in DMEM low glucose medium (HiMedia) for a period of 24 h. Next, the cells were treated with 10 nM 17-α hydroxy-progesterone caproate (progesterone) (MP Biomedicals) in the same medium for 6 h. In case of mifepristone + progesterone (M + P) combination treatment, 100 nM RU486 (mifepristone) was added for 2 h followed by 10 nM progesterone treatment for 6 h in the same medium. An equal volume of alcohol was used as vehicle control.

### Protein sample preparation

Cells were grown to a 70–80% confluence in a 100 mm culture dish and washed thoroughly with sterile 1× PBS. Next, the cells were subjected to 24 h serum starvation (using DMEM low glucose phenol-red free medium) followed by progesterone treatment for 8 h. Alcohol was used as vehicle control. After progesterone treatment, the cells were harvested using a sterile cell scraper and cell lysates were prepared in RIPA Buffer (Sigma-Aldrich) supplemented with a protease-inhibitor cocktail solution (Sigma-Aldrich) and 0.1 M DTT. After intermittently tapping and vortexing the samples on ice, cell debris was pelleted by centrifugation at 14000 rpm after which the protein concentrations were determined using BCA reagent (MP Biomedicals). Bovine serum albumin was used as a standard and the estimations were performed in triplicate.

### Phospho-kinase activation profiling

Kinase activation profiling of T47D (PR-positive) and MDA-MB-231 (PR-negative) breast cancer-derived cells was performed using a Human Phospho-kinase array kit (ARY003B; R&D Systems) according to the manufacturer’s instructions. Briefly, cells were grown in T75 flasks till 70–80% confluence was reached, serum-starved for another 24 h (in serum-free phenol-red-free DMEM medium) and treated with progesterone for 8 h. Next, the cells were harvested, washed with 1× PBS and lysed, after which 400 μg protein from untreated and progesterone-treated samples was incubated overnight at 4 °C with a pre-blocked antibody array nitrocellulose membrane. Subsequently, the membranes were incubated with detection antibodies and probed using streptavidin-HRP, after which signals were developed using chemi-reagents provided with the kit. Exposures to X-ray films were taken from 10 s to 10 min (till saturation was reached). Signal densities of reference spots on both membranes were compared between each pair of membranes used for the control and progesterone-treated samples. The pixel density of each spot, in duplicate, was calculated using ImageJ Array Analyzer plugin. The average pixel density for the duplicate spots for each of the kinases was subtracted from the negative control density. The average pixel densities for control and progesterone-treated samples were plotted as percent phosphorylation for each phospho-kinase. The differential phosphorylation cut-off value was set at 20% increase or decrease in phosphorylation of kinases in response to progesterone.

### Western blotting

Equal amounts of cell lysate were resolved by 10% SDS-PAGE and transferred to PVDF membranes using a wet transfer method. Primary antibodies directed against p-EGFR (Y1068) (Cell Signaling, 3777S; Dilution 1:500), p-AKT (S473) (Cell Signaling, 4060S; Dilution 1:500), p-ERK1/2 (T202/Y204) (Cell Signaling, 9101S; Dilution 1:1000), total EGFR (1005) (Santa Cruz Biotechnology, sc-03; Dilution 1:1000), total AKT (11E7) (Cell Signaling, 4685S; Dilution 1:1000), total ERK2 (c-14) (Santa Cruz Biotechnology, sc-154; Dilution 1:1000) and β-actin (I-19)-R (Santa Cruz Biotechnology, sc-1616-R; Dilution 1:3000) were diluted in 3% BSA solution prepared in 1× TBST and incubated over night at 4 °C. A goat anti-rabbit IgG-HRP secondary antibody (Santa Cruz Biotechnology, sc-2004; Dilution 1:3000) was used for the detection of primary antibody binding. ECL Western Blotting Substrate (Pierce) and Takara Chemiluminescence substrate (ClonTech Takara) were used for visualization of the protein bands on X-ray films (Fuji Films).

### RNA extraction and real-time PCR

For RNA extraction from alcohol and progesterone-treated breast cancer-derived cells, the respective cells were treated with progesterone for 6 h. Next, TRIzol reagent (Invitrogen) was used to lyse the cells after which RNA was isolated according to the manufacturer’s protocol. RNA concentrations were measured using NanoDrop. For assessment of the *DUSP1* transcript levels, cDNA was synthesized using a High capacity cDNA reverse transcription kit (Applied Biosystems) and subjected to quantitative real-time PCR using a Roche Light-Cycler-II 480 instrument in conjunction with a Roche real-time master mix (Roche). Expression changes were calculated using the 2^-ΔΔCT^ method. *GAPDH* was used as internal control for normalization. The primer sequences used for *DUSP1* were Forward primer OAD-571: CCTGCAGTACCCCACTCTACG; Reverse primer OAD-572: CCCAAGGCATCCAGCATGTCC and for *GAPDH* Forward primer OAD-328: AATCCCATCACCATCTTCCA; Reverse primer OAD-329: TGGACTCCACGACGTACTCA.

### Cell invasion assay

A Matrigel invasion assay was performed using 24-well Transwell inserts (Corning) coated with 100 μg matrigel and allowed to settle for 24 h at 37 °C. Next, 35,000 cells suspended in 350 μl serum-free medium were seeded into the upper chamber and 600 μl of 10% serum-containing medium was added to the lower chamber. After this, the cells were allowed to invade for 16-18 h at 37 °C, followed by fixation of the invaded cells and staining by crystal violet. After mounting the membrane using DPX on a slide, the cells were observed under an upright microscope. Ten random fields were chosen after which the number of cells in each field was counted and plotted as percentage cell invasion.

### Scratch wound healing assay

Confluent cell monolayers in 6-well plates were subjected to a scratch with a sterile pipette tip. After this, the cells were briefly rinsed using 1× PBS to remove debris and subsequently incubated with low-glucose phenol-red free DMEM medium containing 10% charcoal-stripped FBS (Gibco). The cells were treated with 10 nM progesterone or 100 nM mifepristone or a combination of both. Alcohol was used as a vehicle control. Cell migration at the wound surface was measured during a period of 20 h under an inverted microscope. Quantification was performed using the ImageJ wound healing plugin tool by measuring the distance of the wound edge of the migrating cells from the start point to the migrated point in three separate wounds in three independent experiments.

## Results and discussion

**Fig. 1:**
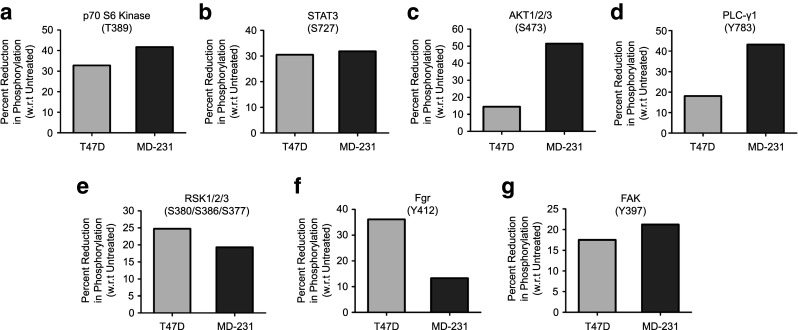
Kinase phosphorylation is modulated by progesterone in breast cancer cells. The percentage of reduction in phosphorylation in response to progesterone was calculated relative to that in untreated cells and is plotted for each of the differentially phosphorylated kinases (panels **a**-**g**). In the bar plot the light grey bar indicates phosphorylation reduction in T47D cells and the dark grey bar indicates phosphorylation reduction in MDA-MB-231 cells

The activation of kinases like EGFR and ERK1/2 has been reported to play an important role in the de-regulation of cellular processes that are associated with the metastatic capacity of breast cancer cells [16]. Here, we set out to assess the effect of progesterone on the activation of kinases in breast cancer cells using a human phospho-kinase array platform. To verify the effect of progesterone independent of the progesterone receptor (PR) status of the cells, we selected both PR-positive (T47D) and PR-negative (MDA-MB-231) breast cancer-derived cells for our study (Table 1). Untreated cells were used as negative controls. As reported before, we observed a breast cancer cell-specific phosphorylation of p53 (S392/S46/S15) and AMPK (T183), which were subsequently used as internal positive controls [17, 18]. Based on differential phosphorylation analyses of the T47D and MDA-MB-231 cells, 7 out of 43 kinases tested were found to be de-phosphorylated in the progesterone treated cells (Fig. 1a-g and Supplementary Fig. 1). Of these, p70 S6 kinase and STAT3 showed the highest decrease in phosphorylation (30%) while FAK, AKT and RSK1/2/3 showed a 20% decrease in both the cell lines in response to progesterone treatment. In addition, we observed a reduction in phosphorylation of the ERK1/2 (T202/Y204, T185/Y187), EGFR (Y1068), MSK1/2 (S376/S360), p38α (T180/Y182) and p27 (T198) kinases upon treatment with progesterone (Supplementary Fig. 1), as reported earlier [19], and validated the results by Western blot analysis (Supplementary Fig. 2a). Consistent with earlier reports [19], we also observed a significant up-regulation of a dual specificity phosphatase, DUSP1, upon treatment with progesterone in breast cancer cells that could possibly mediate the effect observed (Supplementary Fig. 2b). Taken together, our results indicate that progesterone can reduce the phosphorylation of 12 out of 43 kinases tested in a PR-independent manner, which could affect cellular signaling pathways downstream to these kinases with a concomitant increase in the expression of a dual-specificity phosphatase, DUSP1, that could mediate the de-phosphorylation of these kinases [19].

**Table 1:** Selection of breast cell lines and validation of PR/ER/Her2 hormone receptor status. A panel of breast cancer cell lines with varying receptor statuses, as reported in the literature, was selected for studying the effects of progesterone. The validation status of PR/ER/Her2 transcript expression in the cell lines is indicated as “+” (positive) or “-” (negative)

Sr. No.	Cell Line	Literature reported receptor status			Validation of receptor status by RT-PCR		
		PR	ER	HER2	PR	ER	HER2
1.	BT474	+	+	+	+	+	+
2.	T47D	+	+	−	+	+	−
3.	MCF7	+	+	−	+	+	−
4.	ZR-75-1	−	+	−	−	+	−
5.	MDA-MB-231	−	−	−	−	−	−
6.	BT-549	−	−	−	−	−	−

**Fig. 2:**
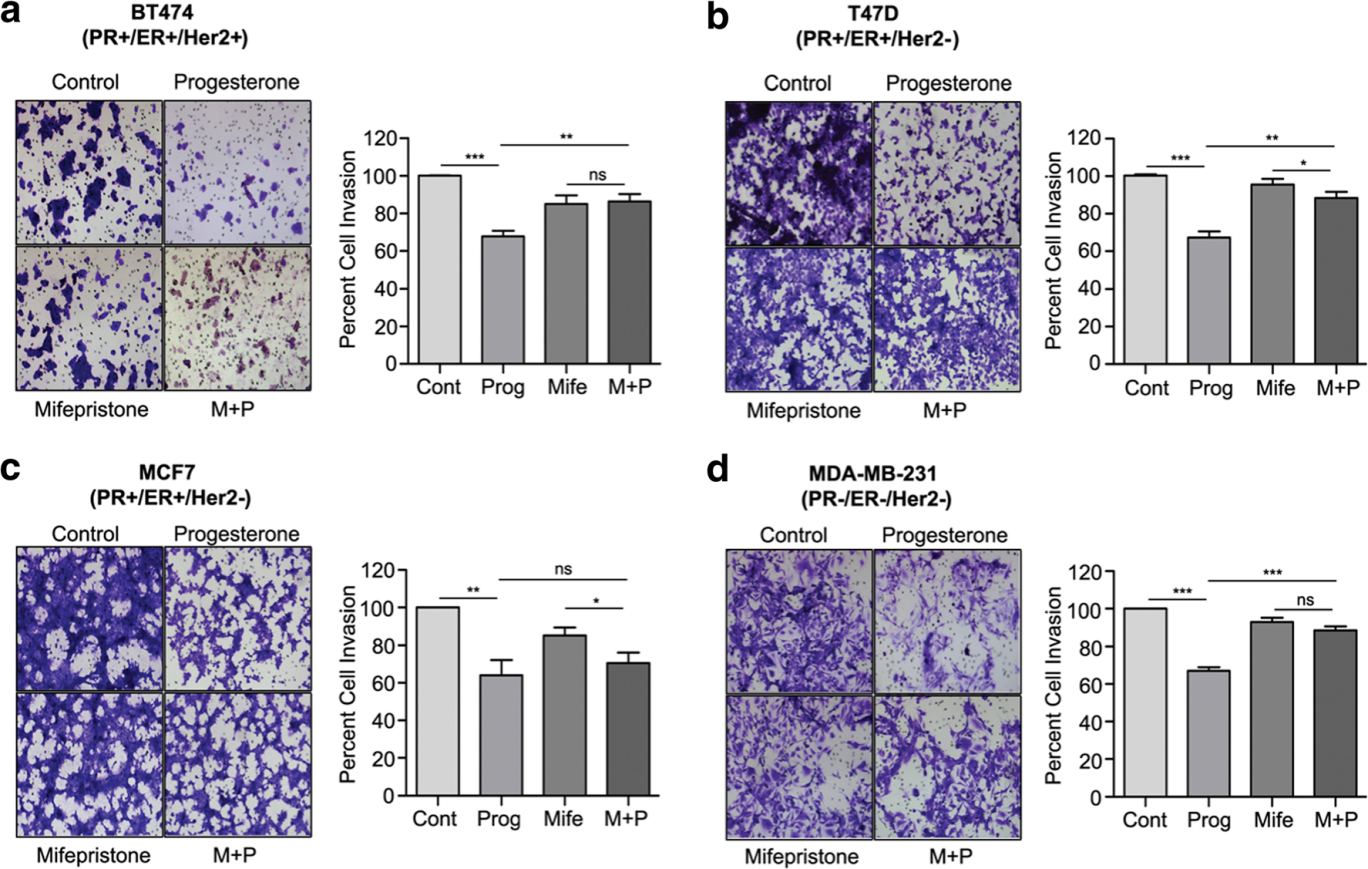
Progesterone inhibits breast cancer cell invasion. Invasion assays were performed with breast cancer-derived cell lines (**a**) BT474, (**b**) T47D, (**c**) MCF7 and d) MDA-MB-231 treated with progesterone, mifepristone or a combination of mifepristone and progesterone (M + P). The bar plot represents the percentage of cell invasion for each panel. The figure is representative of three independent experiments performed in triplicates. *P*-values were calculated using student’s unpaired t-test. ** *p* < 0.001; *** *p* < 0001; ns not significant

Based on the known involvement of EGFR, AKT and ERK1/2 in the invasion and migration of breast cancer cells and the finding that their inhibition may block this phenotype [11, 12, 20] or vice versa, i.e., metastases inhibitors may inhibit the phosphorylation of FAK in PR-negative MDA-MB-231 cells [21] or lung cancer cells [22], we set out to analyze the in vitro effects of progesterone on breast cancer cells. Using a Matrigel chamber assay in conjunction with cells with varying PR/ER/Her2 statuses (Table 1) we found that progesterone could decrease the invasion capacity of different breast cancer-derived cells (BT474, T47D, MCF7 and MDA-MB-231), irrespective of their hormone receptor status (Fig. 2a-d). This result suggests that targeted activation of kinases by progesterone may bring about phenotypic changes in breast cancer-derived cells independent of their PR-status, potentially decreasing their metastatic capacity similar to combinatorial EGFR and AKT inhibition, which is known to affect the invasion of breast and other cancer cells mediated by matrix metalloproteinases [13, 23]. Importantly, we found that PR blocking by mifepristone had no significant effect on the invasive capacities of the cells, again suggesting that progesterone may induce suppression of invasion in breast cancer cells in a predominantly PR-independent manner (Fig. 2a-d).

**Fig. 3:**
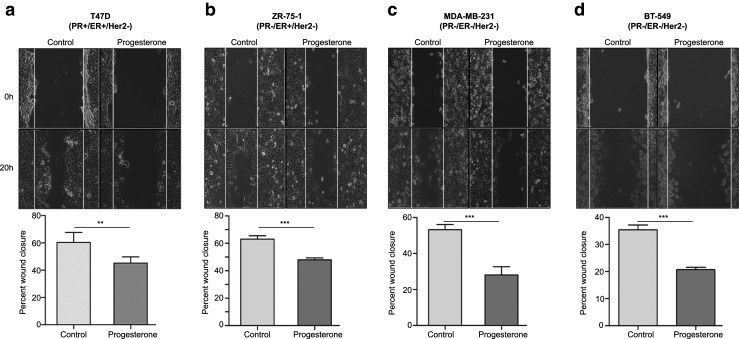
Migration of breast cancer cells decreases in response to progesterone treatment. Scratch wounds were made in breast cancer-derived cell lines (**a**) T47D, (**b**) ZR-75-1, (**c**) MDA-MB-231 and (**d**) BT-549, with differing receptor statuses. Subsequently, the cells were treated with alcohol (control) and progesterone for 20 h and followed in time for migration. The bar plots indicate the percentages of cellular migration, with direct comparisons between control and progesterone treated cells. The figures are representative of three independent experiments performed in triplicates. *P*-values were calculated using student’s unpaired t-test. ** *p* < 0.001; *** *p* < 0.0001

Next we performed scratch wound healing assays to assess whether breast cancer cell migration is affected by progesterone. Similar to the effect of progesterone on breast cancer cell invasion, we observed a significant decrease in cellular migration in response to progesterone over the period of 20 h in a PR-independent manner (Fig. 3a-d). The non-essential role of PR that we observed in the inhibition of migration of breast cancer-derived cells in response to progesterone, specifically in the PR-negative MDA-MB-231, ZR-75-1 and BT-549 cells, may be mediated by interaction of progesterone with the glucocorticoid receptor (GR) or the membrane progesterone receptor (mPR), as has been reported before [22, 24, 25]. Of note, it has also been reported that treatment with glucocorticoids may similarly decrease the migration of PR-negative MDA-MB-231 cells [26], which suggests that redundant pathways may underlie the progesterone response in a PR-independent manner [27]. Consistent with these observations, we found that blocking PR by mifepristone prior to exposing the cells to progesterone did not rescue the effect of progesterone, suggesting that the progesterone-mediated suppression of migration in breast cancer cells is predominantly mediated in a PR-independent manner (Supplementary Fig. 3a and b). This result corroborates a clinical study in which it was found that progesterone may reduce the recurrence of node-positive breast cancer in patients independent of their PR status [3]. A recent in vitro study, however, suggested that the PR status may play an essential role as no significant effect was observed in PR-negative MDA-MB-231 cells in response to R5020, which is a synthetic progestin [28]. But, it has also been shown that the downstream effects of progesterone and progestin may be variable [29] and this notion, together with possible variations that may occur during cell line passage, could account for the phenotypic differences observed in MDA-MB-231 cells. Moreover, we found that the progesterone-mediated suppression of migration and invasion also occurred in other PR-negative breast cancer-derived cells, i.e., ZR-75-1 and BT-549, which has not been reported before.

In summary, we present a first lead to model a randomized in vitro study to systematically elucidate the role of kinases that may underlie the clinical outcome of pre-operative progesterone intervention in breast cancer patients.

## Electronic supplementary material

ESM 1 (PDF 6613 kb)

## References

[1] B. Weigelt, J.L. Peterse, L.J. van't Veer, Breast cancer metastasis: Markers and models. Nat Rev Cancer **5**, 591–602 (2005) 16056258 10.1038/nrc1670

[2] R.W. Carlson and I.C. Henderson, Sequential hormonal therapy for metastatic breast cancer after adjuvant tamoxifen or anastrozole. Breast Cancer Res. Treat. **80** Suppl 1, S19–26; discussion S27–18 (2003) 10.1023/a:1025459232293 14535531

[3] R. Badwe, R. Hawaldar, V. Parmar, M. Nadkarni, T. Shet, S. Desai, S. Gupta, R. Jalali, V. Vanmali, R. Dikshit, I. Mittra, Single-injection depot progesterone before surgery and survival in women with operable breast cancer: A randomized controlled trial. J Clin Oncol **29**, 2845–2851 (2011) 21670457 10.1200/JCO.2010.33.0738

[4] O.J. Scully, B.-H. Bay, G. Yip, Y. Yu, Breast cancer metastasis. Cancer Genomics-Proteomics **9**, 311–320 (2012) 22990110

[5] D. Wolczyk, M. Zaremba-Czogalla, A. Hryniewicz-Jankowska, R. Tabola, K. Grabowski, A.F. Sikorski, K. Augoff, TNF-alpha promotes breast cancer cell migration and enhances the concentration of membrane-associated proteases in lipid rafts. Cell Oncol **39**, 353–363 (2016) 10.1007/s13402-016-0280-x PMC4972855 27042827

[6] H. Mohammed, I.A. Russell, R. Stark, O.M. Rueda, T.E. Hickey, G.A. Tarulli, A.A. Serandour, S.N. Birrell, A. Bruna, A. Saadi, S. Menon, J. Hadfield, M. Pugh, G.V. Raj, G.D. Brown, C. D'Santos, J.L. Robinson, G. Silva, R. Launchbury, C.M. Perou, J. Stingl, C. Caldas, W.D. Tilley, J.S. Carroll, Progesterone receptor modulates ERalpha action in breast cancer. Nature **523**, 313–317 (2015) 26153859 10.1038/nature14583 PMC4650274

[7] H. Singhal, M.E. Greene, G. Tarulli, A.L. Zarnke, R.J. Bourgo, M. Laine, Y.F. Chang, S. Ma, A.G. Dembo, G.V. Raj, T.E. Hickey, W.D. Tilley, G.L. Greene, Genomic agonism and phenotypic antagonism between estrogen and progesterone receptors in breast cancer. Sci Adv **2**, e1501924 (2016) 27386569 10.1126/sciadv.1501924 PMC4928895

[8] H.C. Wang, W.S. Lee, Molecular mechanisms underlying progesterone-enhanced breast cancer cell migration. Sci Rep **6** (2016) 10.1038/srep31509 PMC4980668 27510838

[9] M. Dowsett, I.E. Smith, Presurgical progesterone in early breast cancer: So much for so little? J Clin Oncol **29**, 2839–2841 (2011) 21670461 10.1200/JCO.2011.36.1295

[10] P.S. Steeg, Targeting metastasis. Nat Rev Cancer **16**, 201–218 (2016) 27009393 10.1038/nrc.2016.25 PMC7055530

[11] Z. Yang, R. Bagheri-Yarmand, R.A. Wang, L. Adam, V.V. Papadimitrakopoulou, G.L. Clayman, A. El-Naggar, R. Lotan, C.J. Barnes, W.K. Hong, R. Kumar, The epidermal growth factor receptor tyrosine kinase inhibitor ZD1839 (Iressa) suppresses c-Src and Pak1 pathways and invasiveness of human cancer cells. Clin Cancer Res **10**, 658–667 (2004) 14760089 10.1158/1078-0432.ccr-0382-03

[12] W. Li, Z. Liu, C. Zhao, L. Zhai, Binding of MMP-9-degraded fibronectin to beta6 integrin promotes invasion via the FAK-Src-related Erk1/2 and PI3K/Akt/Smad-1/5/8 pathways in breast cancer. Oncol Rep **34**, 1345–1352 (2015) 26134759 10.3892/or.2015.4103

[13] Y.J. Chen, K.N. Lin, L.M. Jhang, C.H. Huang, Y.C. Lee, L.S. Chang, Gallic acid abolishes the EGFR/Src/Akt/Erk-mediated expression of matrix metalloproteinase-9 in MCF-7 breast cancer cells. Chem Biol Interact **252**, 131–140 (2016) 27087131 10.1016/j.cbi.2016.04.025

[14] Z. Piperigkou, P. Bouris, M. Onisto, M. Franchi, D. Kletsas, A.D. Theocharis, N.K. Karamanos, Estrogen receptor beta modulates breast cancer cells functional properties, signaling and expression of matrix molecules. Matrix Biol **56**, 4–23 (2016) 27179695 10.1016/j.matbio.2016.05.003

[15] J. Kao, K. Salari, M. Bocanegra, Y.L. Choi, L. Girard, J. Gandhi, K.A. Kwei, T. Hernandez-Boussard, P. Wang, A.F. Gazdar, J.D. Minna, J.R. Pollack, Molecular profiling of breast cancer cell lines defines relevant tumor models and provides a resource for cancer gene discovery. PLoS One **4**, e6146 (2009) 19582160 10.1371/journal.pone.0006146 PMC2702084

[16] J. Whyte, O. Bergin, A. Bianchi, S. McNally and F. Martin, Key signalling nodes in mammary gland development and cancer. Mitogen-activated protein kinase signalling in experimental models of breast cancer progression and in mammary gland development. Breast Cancer Res **11**, 209 (2009) 10.1186/bcr2361 PMC2790844 19818165

[17] S. Dutta, C. Warshall, C. Bandyopadhyay, D. Dutta, B. Chandran, Interactions between exosomes from breast cancer cells and primary mammary epithelial cells leads to generation of reactive oxygen species which induce DNA damage response, stabilization of p53 and autophagy in epithelial cells. PLoS One **9**, e97580 (2014) 24831807 10.1371/journal.pone.0097580 PMC4022578

[18] O.S. El-Masry, B.L. Brown, P.R. Dobson, Effects of activation of AMPK on human breast cancer cell lines with different genetic backgrounds. Oncol Lett **3**, 224–228 (2012) 22740885 10.3892/ol.2011.458 PMC3362498

[19] C.C. Chen, D.B. Hardy, C.R. Mendelson, Progesterone receptor inhibits proliferation of human breast cancer cells via induction of MAPK phosphatase 1 (MKP-1/DUSP1). J Biol Chem **286**, 43091–43102 (2011) 22020934 10.1074/jbc.M111.295865 PMC3234857

[20] J.T. Price, T. Tiganis, A. Agarwal, D. Djakiew, E.W. Thompson, Epidermal growth factor promotes MDA-MB-231 breast cancer cell migration through a phosphatidylinositol 3′-kinase and phospholipase C-dependent mechanism. Cancer Res **59**, 5475–5478 (1999) 10554021

[21] G.S. Wu, Y.L. Song, Z.Q. Yin, J.J. Guo, S.P. Wang, W.W. Zhao, X.P. Chen, Q.W. Zhang, J.J. Lu, Y.T. Wang, Ganoderiol A-enriched extract suppresses migration and adhesion of MDA-MB-231 cells by inhibiting FAK-SRC-paxillin cascade pathway. PLoS One **8**, e76620 (2013) 24204647 10.1371/journal.pone.0076620 PMC3812178

[22] M. Xie, S. You, Q. Chen, X. Chen, C. Hu, Progesterone inhibits the migration and invasion of A549 lung cancer cells through membrane progesterone receptor alpha-mediated mechanisms. Oncol Rep **29**, 1873–1880 (2013) 23467643 10.3892/or.2013.2336

[23] D. Kim, S. Kim, H. Koh, S.O. Yoon, A.S. Chung, K.S. Cho, J. Chung, Akt/PKB promotes cancer cell invasion via increased motility and metalloproteinase production. FASEB J **15**, 1953–1962 (2001) 11532975 10.1096/fj.01-0198com

[24] K. Lei, L. Chen, E.X. Georgiou, S.R. Sooranna, S. Khanjani, J.J. Brosens, P.R. Bennett, M.R. Johnson, Progesterone acts via the nuclear glucocorticoid receptor to suppress IL-1beta-induced COX-2 expression in human term myometrial cells. PLoS One **7**, e50167 (2012) 23209664 10.1371/journal.pone.0050167 PMC3509141

[25] M. Xie, L. Zhou, X. Chen, L.O. Gainey, J. Xiao, M.S. Nanes, A. Hou, S. You, Q. Chen, Progesterone and Src family inhibitor PP1 synergistically inhibit cell migration and invasion of human basal phenotype breast cancer cells. Biomed Res Int **2015**, 426429 (2015) 26075237 10.1155/2015/426429 PMC4449873

[26] E.R. Fietz, C.R. Keenan, G. Lopez-Campos, Y. Tu, C.N. Johnstone, T. Harris, A.G. Stewart, Glucocorticoid resistance of migration and gene expression in a daughter MDA-MB-231 breast tumour cell line selected for high metastatic potential. Sci Rep **7**, 43774 (2017) 28262792 10.1038/srep43774 PMC5338339

[27] M.S. Fernandes, J.J. Brosens, B. Gellersen, Honey, we need to talk about the membrane progestin receptors. Steroids **73**, 942–952 (2008) 18221973 10.1016/j.steroids.2007.12.004

[28] C. Bellance, J.A. Khan, G. Meduri, A. Guiochon-Mantel, M. Lombes, H. Loosfelt, Progesterone receptor isoforms PRA and PRB differentially contribute to breast cancer cell migration through interaction with focal adhesion kinase complexes. Mol Biol Cell **24**, 1363–1374 (2013) 23485561 10.1091/mbc.E12-11-0807 PMC3639048

[29] H. Seeger, D. Wallwiener, A.O. Mueck, The effect of progesterone and synthetic progestins on serum- and estradiol-stimulated proliferation of human breast cancer cells. Horm Metab Res **35**, 76–80 (2003) 12734785 10.1055/s-2003-39061

