# Supplementary material for: Progesterone suppresses the invasion and migration of breast cancer cells irrespective of their progesterone receptor status - a short report
Source: Cell Oncol (Dordr). 2017 Jun 26;40(4):411–7. doi: 10.1007/s13402-017-0330-z (PMC5537311; doi:10.1007/s13402-017-0330-z)

Progesterone Suppresses Invasion and Migration of Breast Cancer Cells Independent of Progesterone Receptor

Mukul Godbole[1,2], Kanishka Tiwary[1], Rajendra Badwe[3*], Sudeep Gupta[4*], Amit Dutt[1,2*]

1- Integrated Genomics Laboratory, Advanced Centre for Treatment, Research and Education In Cancer, Tata Memorial Centre, Navi Mumbai- 410210, India.

2- Homi Bhabha National Institute, Training School Complex, Anushakti Nagar, Mumbai, India

3- Department of Surgical Oncology, Tata Memorial Hospital, Tata Memorial Centre, Mumbai- 400012, India.

4- Department of Medical Oncology, Tata Memorial Centre, Mumbai- 400012, India.

* To whom correspondence should be addressed: Amit Dutt adutt@actrec.gov.in; Sudeep Gupta sudeep.gupta@actrec.gov.in; Rajendra Badwe badwera@tmc.gov.in

<u>**Figure Captions**</u>

Supplementary Fig 1 Differentially phosphorylated kinases in response to progesterone treatment of breast cancer cells

The figure represents X-ray films exposed to nitrocellulose membrane for 5min with untreated and progesterone-treated lysates from **a)** T47D and **b)** MDA-MB-231 cells. Black lines and numbers on the right of the spots indicate the location of differentially phosphorylated kinases in untreated and progesterone-treated sets for both cells. The annotation of the spots is as follows:

1- p38α; **2**- ERK1/2; **3**- EGFR; **4**- MSK1/2; **5**- AKT1/2/3; **6**- Fgr; **7**- FAK; **8**- p70S6K;
9- p27; **10**- STAT3; **11**- RSK1/2/3; **12**- PLC-γ1.

Supplementary Fig 2 Progesterone suppresses phosphorylation of kinases involved in cell migration and invasion in breast cancer cells by up-regulating *DUSP1*

a) Western blot analysis of p-EGFR (Y1086), p-AKT (S473) and p-ERK1/2 (T202/Y204) was performed in T47D and MDA-MB-231 (MD231) cells treated with progesterone. Total protein for each phospho-kinase was also probed. Numbers on blot indicate ratio of intensity of phosphorylation for each kinase with respect to its total protein levels. β-actin was used as internal loading control. β-actin for the p-ERK1/2 and p-EGFR panels is the same. "-" indicates control while "+" indicates progesterone treatment. **b)** Real-time PCR analysis of *DUSP1* was performed in breast cancer cells treated with progesterone. Graph has been plotted as fold-change for *DUSP1* with respect to *GAPDH* for control and progesterone-treated cells; horizontal black line indicates expression of *DUSP1* in control cells. Figure is representative of three independent experiments performed in triplicates. *P*-value was calculated using student's unpaired t-test. ** indicates *P*-value <0.001; *** indicates *P*-value <0.0001.

Supplementary Fig 3 Mifepristone antagonizes the effect of progesterone on cell migration

a) T47D and **b)** MDA-MB-231 cells were treated with alcohol (control), progesterone, mifepristone and mifepristone+progesterone for 20hrs and followed for time-lapse cellular migration assay. Bar plots indicate percentage cellular migration of the cells, with a comparison between control and progesterone; mifepristone and mifepristone+progesterone; and progesterone and mifepristone+progesterone treated cells. Figures are representative of three independent experiments performed in triplicates. *P*-value was calculated using student's unpaired t-test. ** indicates *P*-value<0.001; *** indicates *P*-value<0.0001.

Supplementary Fig 1

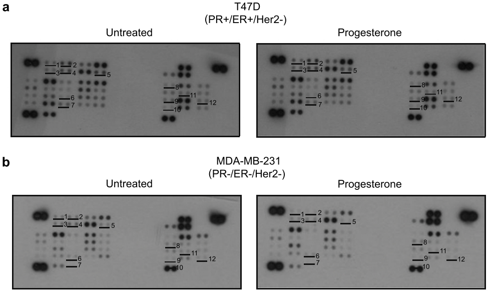

Supplementary Fig 2

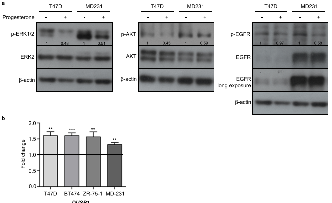

Supplementary Fig 3

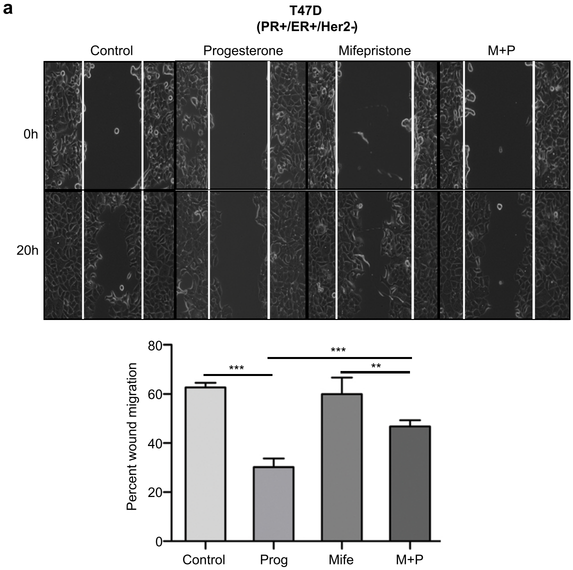

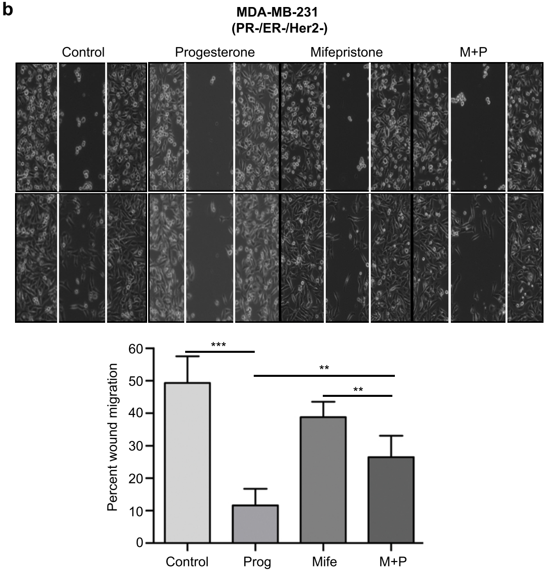

Supplement: Supplementary file 1 — (PDF 6613 kb) [file 13402_2017_330_MOESM1_ESM.pdf]
